# Modelling Marek's Disease Virus (MDV) infection: parameter estimates for mortality rate and infectiousness

**DOI:** 10.1186/1746-6148-7-70

**Published:** 2011-11-11

**Authors:** Katherine E Atkins, Andrew F Read, Nicholas J Savill, Katrin G Renz, Stephen W Walkden-Brown, Mark EJ Woolhouse

**Affiliations:** 1 Centre for Infectious Diseases, University Of Edinburgh, West Mains Road, EH9 3JT, UK; 2 Center for Infectious Disease Dynamics, Departments of Biology and Entomology, 208 Mueller Laboratory, The Pennsylvania State University, University Park, PA 16802, USA; 3 Centre for Animal Health and Welfare, School of Environmental and Rural Science, The University of New England, Armidale NSW 2351, Australia; 4 Department of Epidemiology and Public Health, Yale School of Medicine, New Haven, CT 06510

## Abstract

**Background:**

Marek's disease virus (MDV) is an economically important oncogenic herpesvirus of poultry. Since the 1960s, increasingly virulent strains have caused continued poultry industry production losses worldwide. To understand the mechanisms of this virulence evolution and to evaluate the epidemiological consequences of putative control strategies, it is imperative to understand how virulence is defined and how this correlates with host mortality and infectiousness during MDV infection. We present a mathematical approach to quantify key epidemiological parameters. Host lifespan, virus latent periods and host viral shedding rates were estimated for unvaccinated and vaccinated birds, infected with one of three MDV strains. The strains had previously been pathotyped to assign virulence scores according to pathogenicity of strains in hosts.

**Results:**

Our analyses show that strains of higher virulence have a higher viral shedding rate, and more rapidly kill hosts. Vaccination enhances host life expectancy but does not significantly reduce the shedding rate of the virus. While the primary latent period of the virus does not vary with challenge strain nor vaccine treatment of host, the time until the maximum viral shedding rate is increased with vaccination.

**Conclusions:**

Our approach provides the tools necessary for a formal analysis of the evolution of virulence in MDV, and potentially simpler and cheaper approaches to comparing the virulence of MDV strains.

## Background

Marek's Disease Virus (MDV) is an oncogenic poultry herpesvirus of considerable economic importance to the poultry industry. Virus strains have become increasingly virulent since the 1960s [1,2]. The causes of this viral evolution are unclear [3]. Quantification of patterns of viral shedding and virus-induced host mortality are necessary for a rigorous understanding of the epidemiology of a disease, not least to identify increases in virulence. Here we develop methods to do this.

MDV is an airborne pathogen with infection occurring via inhalation [4]. Virus shedding occurs by infected feather follicle epithelium [5]. The resulting dust and dander from dead stratified cells and moulted feathers can then remain in the environment and act as a reservoir for chicken infection. Clinical signs are varied and result in significant morbidity and mortality depending on host genetic susceptibility and virulence of the MDV strain [6]. Symptoms include polyneuritis (an enlargement of multiple peripheral nerves), visceral lymphoma (tumours affecting organs such as the heart, liver, spleen etc.), acute transient paralysis, immunosuppression, brain oedema and acute rash. There has been a change in the types of clinical signs since the disease was first noted [7,8], when chronic polyneuritis was the only sign. Since then, the list of clinical signs described above expanded gradually over the decades [2].

Almost all industrialised countries have experienced MD losses in their poultry industry and a crude estimate of the cost of Marek's disease is said to be in the range of US $1-2 billion annually [7]. Control of Marek's disease is predominantly via vaccination of chickens. Crucially the MDV vaccine was the first vaccine to be developed against any cancer [9]. Three types of vaccine have been developed for use against MD. These are herpesvirus of turkeys (HVT), non-pathogenic serotype 2 MDV and non-pathogenic serotype 1 [10]. These vaccines have been used in different sequences in different countries and the vaccine types have also been commonly combined in bivalent or trivalent vaccines.

Definitions of virulence are numerous and varied [11]. Microbiologists equate virulence with the notions of both infectivity and severity of disease, whereas evolutionary biologists focus on evolutionary fitness of either the pathogen or the host [12]. Zoologists tend to focus on host fitness [13,14] with most mathematical models describing this specifically as host mortality [14,15].

There have been various attempts to define virulence in the context of MDV [16]. For example, Witter [1] relates the percentage of HVT or Bivalent (HVT + serotype 2) vaccinated chickens, that when infected with a particular strain develop gross lesions or die of Marek's disease within eight weeks to the figure in unvaccinated chickens challenged with the same strain. This percentage score uses the protective effect of vaccines as its metric for virulence. In addition, [17] use neurovirulence as a tool for pathotyping MDV strains, noting that many of the very virulent strains are synonymous with high levels of neuropathology. This method circumvents the need for both vaccine-based definitions and therefore high numbers of birds, and also may cut the experiment time. In the first pathotyping regime, there are four recognised MDV serotype 1 pathotypes, each occupying part of the 'continuum of virulence' [1] defined as: mild, m; virulent, v; very virulent, vv; very virulent plus, vv+. This grouping correlates very well with the second pathotyping regime [18].

Control strategies for MDV require an understanding of the epidemiology of the disease, in particular how virulence relates to key parameters such as viral shedding rates and duration of infectious period. There have been efforts to find correlates with virulence of MDV isolates, most notably with viral load (virus within a bird tissue). This has been achieved by cell culture techniques [19] and intra-cellular detection during the first 10 days of infection [20]. However, the relationship between viral load (or replication) and virulence has not been convincingly tested [16]. PCR testing methods have been developed in order to quantify viral loads [20-26], which have enabled the viral loads in shed dust to be directly measured [27,28]. The infectious period is defined as the total time in which an infected bird sheds virus [29]. This is determined by the clearance rate and the lifespan of the individual host. Since there is no recovery from MDV infection, the infectious period is defined by a total of four parameters: the disease-induced mortality, the latent period (the time between infection and infectiousness of a host) and two other non-disease associated parameters, namely non-disease induced mortality and the maximum lifespan of an individual (the farm slaughter time in the case of broilers).

Here we present methods for parameter estimation for MDV isolates, allowing a formal quantification of infected host lifespan, viral shedding rates and viral latent periods. The parameters determined from the analysis allow comparison between isolates of different virulence in both unvaccinated and vaccinated birds. It is anticipated that the parameter estimates will be used for a better understanding of the pathology and aetiology of the disease itself and as a platform for investigation into the causes of virulence evolution.

## Results

In the first section, we used survival analysis with a Weibull mortality function to build a statistical model for the lifespan of birds infected with MDV. In the second section, we developed a dynamic model to simulate the shedding of virus by a group of birds and used Bayesian techniques to estimate the four key infectiousness parameters: primary and secondary latent periods and viral shedding rates.

### Mortality

**Figure 1:**
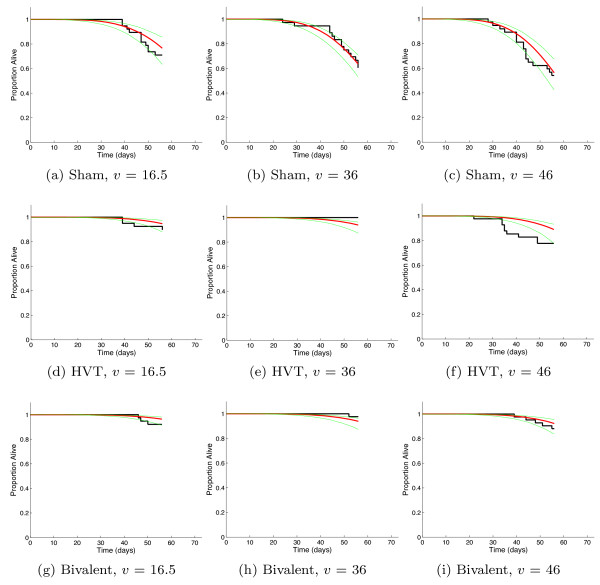
**Mortality: Three different treatments crossed with three different virulence scores as stated**. Graphs show data (black), maximum likelihood estimate of Weibull distribution (red) and 95% credible interval (green). Transformed virulence score (continuous) and vaccine treatment (categorical) are the (additive) covariates.

**Figure 2:**
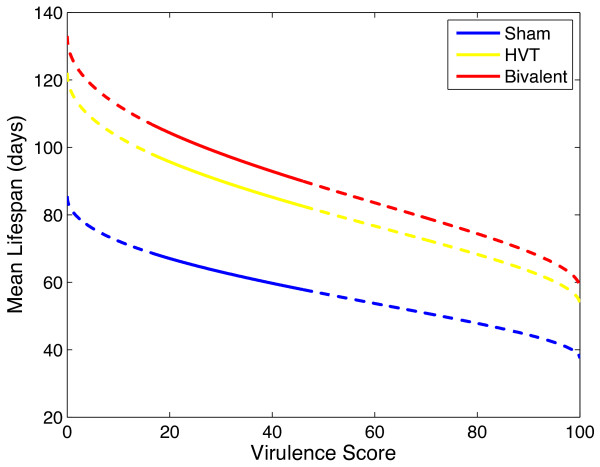
**Mortality: Maximum likelihood estimates of ***λ *and *r *give the mean lifespan $(\lambda \Gamma \left(1+\frac{1}{r}\right)$, **where Γ(*z*) is the Gamma function as it varies with virulence score**. The dashed lines represent extrapolation outside virulence scores given in data.

An additive covariate Weibull regression model was fitted to the bird survival data, since the interaction terms were not significant. The model estimates are given in Table 1. The Weibull model captures the data significantly better with the covariates used than without (*p *< 0.01). The resulting graphs showing the data and Weibull model fit are displayed in Figure 1. The model showed that host lifespan decreased with virulence score and increased with vaccination (Figure 2 and Table 1), although there was no significant difference between the effect of the two vaccines on lifespan (Table 1). On most days there were either zero or one birds dying from MDV-related illness from the initial 52 or 53 individual birds in each group (Figure 1). These data and further information are published in separate experimental work [30].

**Table 1:** Survival analysis: Estimated coefficients of the covariates in the survival analysis.

Symbol	Coefficient	Value (95% CI via McMC)	*z *	*p *
*r*	Shape Parameter	4.18 (3.38,4.99)	-11.60	< 0.001
*β*_0_	Intercept	4.54 (4.23,4.87)	26.08	< 0.001
*β*_1_	Virulence Score	-0.53 (-1.01,-0.06)	-2.04	0.040
*β*_2_	HVT vaccine	0.44 (0.24,0.66)	3.95	< 0.001
*β*_3_	Bivalent vaccine	0.35 (0.19,0.52)	4.28	< 0.001

The transformed virulence score is a continuous covariate and the vaccine treatment is a categorical covariate in an additive regression, giving the test statistic, *z *and the corresponding *p *value. The numbers in brackets are the 95% credible intervals.

### Shedding

**Figure 3:**
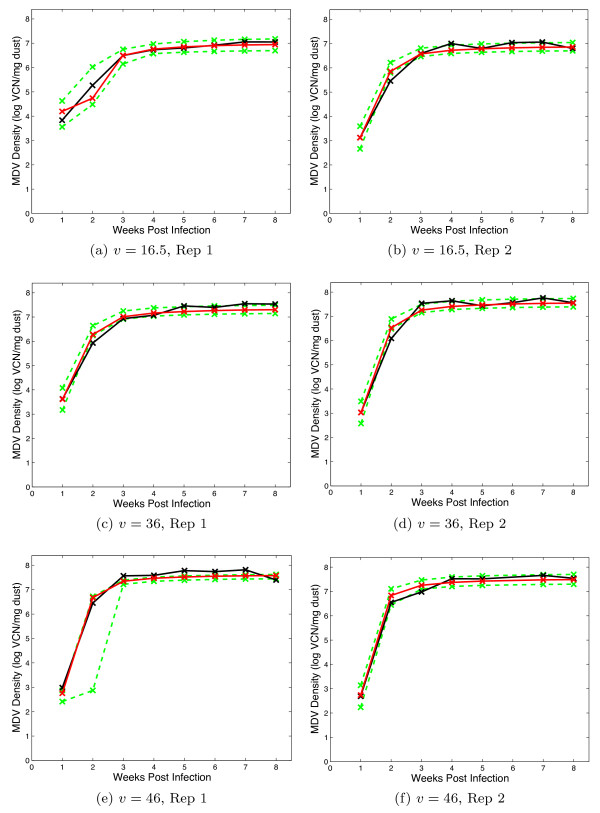
**Viral Shedding: Sham vaccinated treatments**. Graphs include the data (black), the maximum likelihood realisation (red) and the 95% credible interval (green). The panels show each replicate for the virus strains which have been given the virulence score, *v *as denoted in the caption.

**Figure 4:**
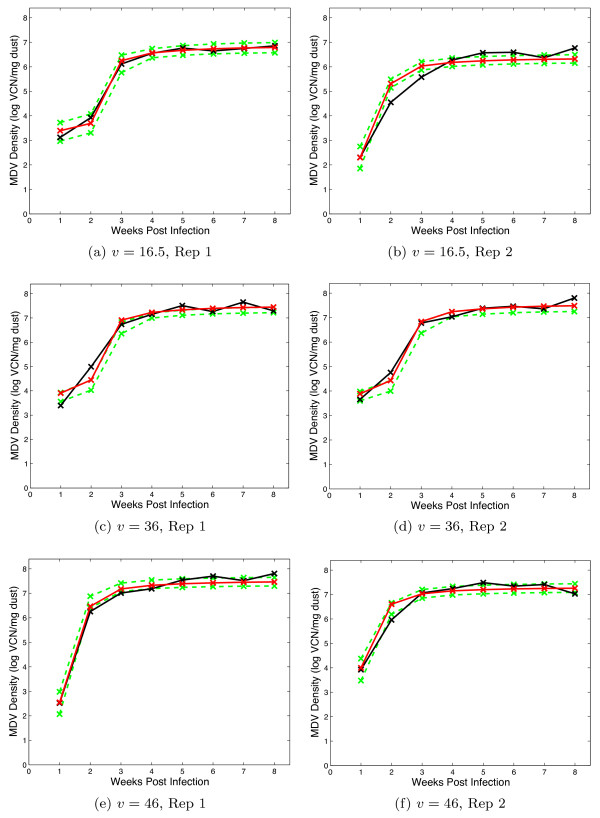
**Viral Shedding: HVT vaccinated treatments**. Graphs include the data (black), the maximum likelihood realisation (red) and the 95% credible interval (green). The panels show each replicate for the virus strains which have been given the virulence score, *v *as denoted in the caption.

**Figure 5:**
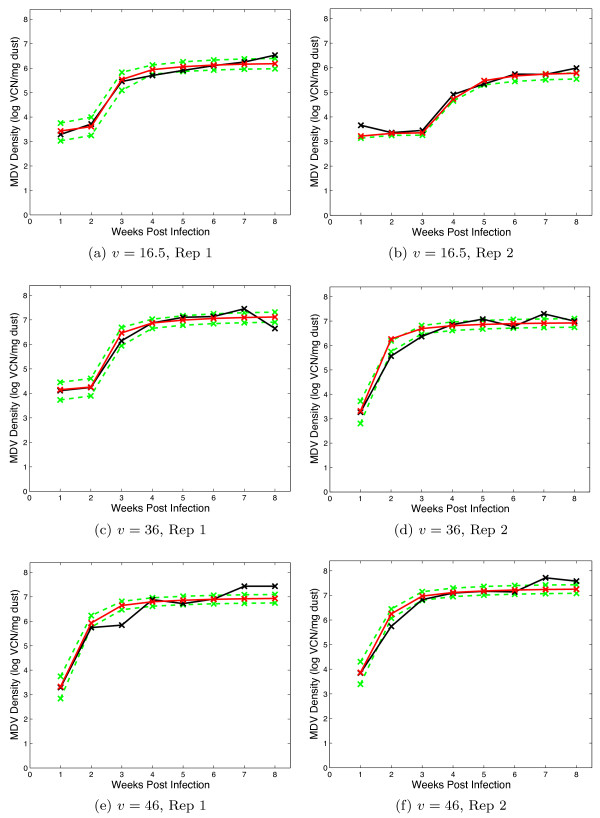
**Viral Shedding: Bivalent vaccinated treatments**. Graphs include the data (black), the maximum likelihood realisation (red) and the 95% credible interval (green). The panels show each replicate for the virus strains which have been given the virulence score, *v *as denoted in the caption.

The dynamic model output records the density of virus (measured in viral copy number (VCN)/mg dust), which fitted to the data from each isolator independently. The model fits can be compared directly in Figures 3, 4 and 5, which give the day of sampling and the sample value. These data and further information are published in a separate experimental paper [30].

For each isolator, the median of the posterior distribution for the four parameters in the model (two latent periods and two rates of viral shedding) are given in Tables 2, 3 and 4, where the number of air changes per hour, *α*, is set to 15. Assuming *α *∈ [5, 25], the results obtained in the McMC method did not change, whatever value of *α *is chosen.

**Table 2:** Viral shedding unvaccinated: median and 95% credible intervals for the posterior distribution of the four parameters for both replicates of the sham vaccinated factorial study for different virulence scores, *v*.

			VIRULENCE SCORE			
PARAMETER	16.5		36		46	
	**Rep 1**	**Rep 2**	**Rep 1**	**Rep 2**	**Rep 1**	**Rep 2**
1 Latent Period (days)	6 (2,6)	4 (0,6)	4 (0,6)	4 (0,6)	3 (0,6)	3 (0,6)
2 Latent Period (days)	10 (7,14)	9 (6,13)	9 (6,13)	9 (6,13)	9 (6,13)	9 (5,13)
1 Shedding Rate (logVCN/mg dust)	4.87 (3.79,5.25)	3.37 (2.79,4.05)	3.86 (3.30,4.56)	3.28 (2.71,3.95)	3.20 (2.64,3.90)	2.89 (2.34,3.59)
2 Shedding Rate (logVCN/mg dust)	6.99 (6.72,7.21)	6.89 (6.72,7.07)	7.34 (7.17,7.51)	7.58 (7.41,7.76)	7.69 (7.51,7.85)	7.52 (7.32,7.72)

The numbers in brackets are the 95% credible intervals.

**Table 3:** Viral shedding HVT vaccination: median and 95% credible intervals for the posterior distribution of the four parameters for both replicates of the HVT vaccinated factorial study for different virulence scores, *v*.

			VIRULENCE SCORE			
PARAMETER	16.5		36		46	
	**Rep 1**	**Rep 2**	**Rep 1**	**Rep 2**	**Rep 1**	**Rep 2**
1 Latent Period (days)	5 (0,6)	2 (0,6)	6 (4,6)	6 (2,6)	3 (0,6)	3 (0,6)
2 Latent Period (days)	12 (8,17)	10 (7,13)	11 (8,14)	12 (8,16)	10 (6,13)	9 (6,13)
1 Shedding Rate (logVCN/mg dust)	3.79 (3.34,4.20)	2.44 (1.93,3.02)	4.58 (4.19,4.92)	4.56 (4.13,4.91)	2.70 (2.16,3.42)	4.15 (3.58,4.82)
2 Shedding Rate (logVCN/mg dust)	6.83 (6.61,7.04)	6.35 (6.18,6.52)	7.49 (7.28,7.70)	7.53 (7.31,7.75)	7.50 (7.32,7.67)	7.29 (7.11,7.46)

The numbers in brackets are the 95% credible intervals.

**Table 4:** Viral shedding bivalent vaccination: median and 95% credible intervals for the posterior distribution of the four parameters for both replicates of the Bivalent vaccinated factorial study for different virulence scores, *v*.

			VIRULENCE SCORE			
PARAMETER	16.5		36		46	
	**Rep 1**	**Rep 2**	**Rep 1**	**Rep 2**	**Rep 1**	**Rep 2**
1 Latent Period (days)	4 (0,6)	2 (0,5)	3 (0,6)	3 (0,6)	3 (0,6)	3 (0,6)
2 Latent Period (days)	14 (9,19)	23 (18,27)	15 (10,19)	9 (6,13)	10 (6,13)	10 (6,13)
1 Shedding Rate (logVCN/mg dust)	4.87 (3.27,5.19)	3.37 (3.27,3.83)	3.86 (2.92,4.70)	3.28 (2.93,4.14)	3.20 (2.94,4.18)	2.89 (2.49,4.69)
2 Shedding Rate (logVCN/mg dust)	6.23 (6.02,6.45)	5.85 (5.61,6.09)	7.16 (6.94,7.37)	6.95 (6.78,7.13)	6.96 (6.78,7.12)	7.28 (7.11,7.45)

The numbers in brackets are the 95% credible intervals.

A linear model was fitted to account for the variation in the primary and secondary latent periods, with the latent period as the response variable and arcsine square-root transformed virulence score and vaccine treatment as multiplicative covariates. For the primary latent period, none of the results were significant (*p *= 0.41), showing that the primary latent period did not differ among virus strains and nor was it altered by vaccine treatment. For the secondary latent period, neither virulence score nor HVT vaccine affected the duration of the latent period, however, Bivalent vaccine increased the duration of the second latent period significantly and to a greater extent for smaller virulence scores (see Table 5).

**Table 5:** Secondary latent period: linear regression results for effect of transformed virulence score and vaccine treatment on the secondary latent period (days).

	Estimate	Std. Error	t value	Pr(> |*t*|)
Intercept	10.16	4.30	2.36	0.04
Virulence Score	-1.64	6.98	-0.24	0:82
VaccHVT	2.60	6.10	0.43	0:68
VaccBiv	19.29	6.10	3.16	< 0.01
Virulence Score:VaccBiv	-24.82	9.88	-2.51	0.03
Virulence Score:VaccHVT	-1.82	9.88	-0.18	0.86

Adjusted *R*^2 ^= 0.54, *p *= 0.01.

**Figure 6:**
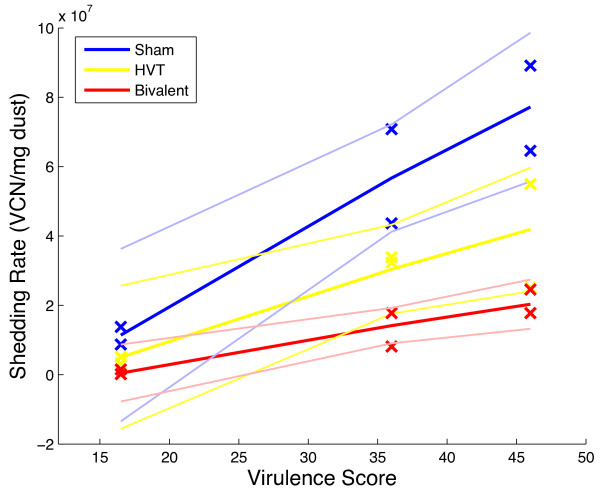
**Viral Shedding: Linear regression analysis of the virulence score and the corresponding estimated secondary viral shedding rate for all treatments**. The 95% confidence intervals for the mean response are given in fainter lines. The linear regression results are shown in Table 6.

An association between the virulence score and the estimated viral shedding rates was found. A linear regression was used to estimate the viral shedding rate as a function of arcsine square-root transformed virulence score and vaccine treatment. There was no significant association between the primary viral shedding rate and HVT or Bivalent vaccine treatment (*p *= 0.085, 0.10 respectively) nor virulence score (*p *= 0.055). There was, however, a strong positive association between the virulence score of a strain and the secondary viral shedding rate (*p *= 0.00076). There was no association between the secondary viral shedding rate and either HVT or Bivalent vaccine treatment (*p *= 0.66, 0.39 respectively, see Table 6). Figure 6 shows the estimated secondary viral shedding rate as a function of virulence score for different vaccine treatments.

**Table 6:** Secondary viral shedding: linear regression results for effect of transformed virulence score and vaccine treatment on the second viral shedding rate (VCN/mg dust).

	Estimate	Std. Error	t value	Pr(> |*t*|)
Intercept	-3.24 × 10^7^	1.35 × 10^7^	-0.24	0.03
Virulence Score	9.79 × 10^7^	2.19 × 10^7^	4.47	< 0.01
VaccHVT	8.51 × 10^6^	1.91 × 10^7^	0.45	0.66
VaccBiv	1.70 × 10^7^	1.91 × 10^7^	0.89	0.39
Virulence Score:VaccBiv	-5.73 × 10^7^	3.19 × 10^7^	-0.19	0.09
Virulence Score:VaccHVT	-2.37 × 10^7^	3.10 × 10^7^	-0.77	0.46

Adjusted *R*^2 ^= 0.74, *p *< 0.01.

## Discussion

The survival model fits the nine survival curves well on the whole, with both replicates combined into one fit. The delay which characterises the mortality profile for Marek's disease is captured well by the Weibull model. In most cases, the viral shedding model captures the data well.

The viral shedding model assumes that all birds become initially infected with MDV. While it is difficult to model individual birds based on the resolution of the data available, we do point out that the assumption that all birds become infected precipitates the estimated per bird viral shedding rates being perhaps an over-estimate of the actual rates. On the other hand, the qPCR methods used estimate total viral copies which is not necessarily equivalent to total infectious material and therefore serves as an upper limit of viable infectious DNA.

Our estimate of the primary latent period of between 2 and 6 days is much shorter than earlier estimates of around 13 days [31] but only slightly shorter than more recent estimates based on new PCR techniques which have detected significant quantities of virus in feather tips [32] and feather dander [27,28] at 7 days post infection. This discrepancy may be due to a number of factors: detectability of virus at low quantities, the sampling time of the experiments and heterogeneities between the experiments. The estimates here also have confidence intervals between 0-6 days which suggest the data are not able to give a great deal of certainty on the exact time of onset of viral shedding. Further analysis might take into account biological knowledge through prior distributions to get a more exact value, and past experimental studies would suggest shedding begins towards the end of the estimated interval [27,28].

The pathotyping of MDV isolates, similar to that pioneered by Witter and colleagues [1,16], is a useful tool for comparing the pathogenicity of strains with respect to others in vaccinated birds. However several of the more recent isolated, highly virulent strains have ranks above 80, in some cases 90 on Witter's ranking system. Should more virulent strains emerge, it will be difficult to categorise them via this method, as the scale is truncated at 100. The method of fitting a Weibull distribution proposed in this paper allows for a greater flexibility of pathotyping, in that an isolate may be categorised according to its mean time to kill the host. The subsequent effect of vaccination of the host on this metric can then be established.

Biologically, it may seem more sustainable to pathotype according to the lifespan of an unvaccinated bird, but experimentally it may also be a solution to the costly procedure of having two or three groups of birds, each vaccinated with a different treatment. Even a simple 'time to death' metric can be performed in relatively few birds, as was the case for the myxoma virus in rabbits in Australia. Myxoma strains were pathotyped effectively when the case fatality rate proved experimentally intensive and ineffective in discriminating between similarly virulent strains [33,17] noted this restriction of current pathotyping regimes, instead favouring a system based on neuropathology to circumvent these methodological and experimental limitations. The analysis presented here differs from both of these past approaches due to its epidemiological focus on virulence. However, we point out that the methodology adopted here only analyses three distinct strains. Therefore, a wider range of strain virulences and places of origin will be required to sufficiently extrapolate the current results to the whole virulence spectrum.

## Conclusions

This paper uses a mathematical approach to estimate key epidemiological parameters for Marek's Disease Virus (MDV). We used survival analysis with a Weibull mortality function to build a statistical model for the lifespan of birds infected with MDV. We were able to compare the relative impact of virulence score and vaccine covariates by calculating credible intervals via Bayesian methods. We developed a dynamic model to simulate the shedding of virus by a group of birds and used Bayesian techniques to estimate the four key infectiousness parameters: primary and secondary latent periods and viral shedding rates.

We applied these methods to the data for three MDV strains varying moderately in virulence. From the survival analysis, we found that increasing the virulence score of a virus strain decreases the lifespan of the host (Figure 2). Vaccinating the host with either the first or second generation vaccine increases its lifespan, but neither one to any greater degree (Table 1). From the viral shedding analysis, we concluded primary latent period is not affected by vaccination or virulence score, however, secondary latent period increases with Bivalent vaccine treatment (Table 5). Secondary (long term) viral shedding rate (measured in VCN per mg dust) is higher for strains of higher virulence (Table 5 and Figure 6). Vaccination does not reduce long term viral shedding rate significantly. Together, these results imply that more virulent viruses shed more infectious material into the environment and Bivalent vaccines reduce the infectious period, especially for less virulent strain infections.

The results described here are qualitatively in line with current thinking on MDV pathogenesis, but the new methods presented allow a formal comparison of strains in an epidemiological meaningful way. It is anticipated that this analysis will allow a more quantitative understanding of the aetiology of MDV and the mechanisms governing a functional rise in virulence for a strain. With this analysis it is hoped that a more epidemiologically significant classification of MDV strains will emerge which will eventually provide not only insight into the reasons for evolution to highly virulent strains but also a metric to evaluate future risks and the efficacy of possible control strategies.

## Methods

### Experimental Data

We look at a single experiment [30] with two response variables measured. Groups of maternal antibody positive IsaBrown layer chicks were inoculated with one of three vaccine treatments: sham (diluent only), HVT (first generation industry vaccine) or Bivalent (second generation industry vaccine) at one day old. At 5 days post vaccination, the birds were challenged with 500 pfu (plaque forming units) of one of three MDV strains: 04CRE, MPF57 and 02LAR. These independently sampled isolates were Australian in origin and have been pathotyped with virulence scores (the percentage of HVT or Bivalent vaccinated chickens infected with a particular strain which develop gross lesions or die of MDV within eight weeks [34]) of 16.5, 36 and 46 respectively. The viruses were all isolated from field outbreaks and used at passage level 4 to 7 in chick kidney cells. The experiment was therefore a 3 × 3 factorial design study, with vaccine type fully cross-factored against virus strain. Each treatment combination was replicated in two separate isolators resulting in 26 or 27 birds per combination for the pathotyping component of the study. The air in each isolator was changed 8-20 times per hour. Further details can be found in [30]. The experiment was approved by the University of New England Animal Ethics Committee with approval number AEC05/076. Due to Animal Ethics regulations, birds thought to be nearing death were euthanised and classified as mortality in the experimental results. The experiment lasted 56 days post infection (dpi) and all birds were humanely killed and examined for MD lesions on the last day. Therefore for the purposes of this analysis, the data were censored after day 55. All birds were given a large dose of MDV (500 pfu) via intra-abdominal injection and it was therefore assumed that all the birds were infected and would shed the virus. Differences between unvaccinated and vaccinated birds in detection of MD have been reported [35], but these have been in studies where infection has been by injection with 50 pfu. Increasing the MDV dose fourfold (from 50 to 200 pfu) significantly increased the detected incidence of MD in unvaccinated birds from 64% to 81% [27].

The two variables recorded were the time to death of a bird (measured in days) and the density of virus within each isolator every week (measured in logVCN per mg of dust in the environment). The virus titres in the dust were determined via quantitative real-time PCR of dust collected from the isolator exhaust ducts, the methods of which have been discussed in separate studies [25]. The data are shown in Figures 1 (mortality), 3, 4 and 5 (shedding).

### Parameter Estimates

We assumed that each infected bird starts shedding virus after an initial delay, then after a further delay, the rate at which it sheds virus rises to a long-term stable value. We estimated five key epidemiological parameters: host lifespan, primary latent period (the time until an infected bird becomes infectious), secondary latent period (the delay between an infectious bird first shedding virus and when it sheds virus at its secondary long term rate) and viral shedding rates (both primary and secondary, measured in VCN/mg dust). A survival analysis was undertaken for the mortality data because multiple covariates were needed to find the host lifespan. These covariates were vaccine treatment and virus virulence score. Latent period and rate of viral shedding could not be estimated directly from the data since the response variable (measured weekly in VCN per mg dust) could vary with the number of birds housed together, which changed as birds died. Therefore a dynamic model capturing this variability was used to estimate the two infectiousness parameters.

For ease of notation, log is written to represent log_10 _throughout the paper.

#### Mortality

The biology of Marek's disease suggests that the probability of death by MDV infection changes through time, since the virus undergoes lytic, latent stage (this is a description of an MDV pathogenesis stage and not equivalent to the 'latent periods' in the epidemiological context which this study estimated), late lytic (where infection is extended to other organs of the host), and transformative (tumour-induction) stages. These stages are pathologically distinct and may lead to a different mortality rate. Therefore, a Weibull distribution was chosen as a candidate distribution for modelling survivorship curves, which is often used for time to death data since it is flexible and can mimic other distributions but only has two parameters in its non-location form. We therefore assumed that time to death can be modelled as a random variable, *T*, such that *T *~ *W*(*r*, *λ*) where *r*, *λ *> 0 are the shape and scale parameters respectively. The associated probability density function is

$$f\left(t\right)=\left\{\begin{array}{cc} \frac{r}{\lambda }{\left(\frac{t}{\lambda }\right)}^{{}^{r-1}}e^{-{\left(\frac{t}{\lambda }\right)}^{r}} & \text{if}\;t\geq 0\\ 0 & \text{if}\;t<0 \end{array}\right)$$

A Weibull model was fitted to the survival data in the study. In the case when *r *= 1, the distribution collapses to an exponential distribution. When *r *> 1 or *r *< 1 there is an increasing or decreasing chance of death over time respectively. Coefficients, ***β***, are such that ***λ ***= exp(***β ***· **x**), where ***β ***= [*β*_0_, *β*_1_, *β*_2_, *β*_3_] are the covariate coefficients and **x **= [1, *x*_1_, *x*_2_, *x*_3_]*^T ^*are the covariates. In this analysis, there were three covariates (one continuous: the transformation of the virulence score of an isolate (*x*_1_) and two binary: the presence or absence of HVT (*x*_2_) and Bivalent (*x*_3_) vaccine). Therefore there were 9 combinations on the set of possible covariates, thus *λ_j _*= ***β****·**x**_j _*where *j *∈ [1, 9] The likelihood function can therefore be written

L(λ,r)=∏j=19∏i=inrλjtiλjr-1δi exp-tiλjr

where *δ_i _*is zero when the *i*th observation is censored and unity elsewhere. This function was maximized via the Newton-Raphson algorithm such that $L\left(\tilde{\lambda },\tilde{r}\right)=\max L\left(\lambda ,r\right)$where $\tilde{\lambda }$ and $\tilde{r}$ are the maximum likelihood estimates. Note that the virulence score, *v*, of an isolate is a percentage measure and to be used as an explanatory variable in a regression analysis, it should be transformed such that $v_{T}=\arcsin\sqrt{0.01v}$. The regression fitted the maximum likelihood estimates for ***β***, however further Bayesian analysis was required to estimate the associated credible intervals when the covariate covariance matrix does not approximate the identity matrix. An McMC framework was set up in WinBUGS [36] to calculate the posterior distributions and the credible intervals of the Weibull survival function.

The prior distributions of each parameter were assumed to be uninformative and were thus taken as uniform, to ensure equivalence to maximum likelihood estimation. The burn-in was set to 22,000 iterations with every 10th sample taken from the subsequent 100,000 iterations [37,38].

#### Viral Shedding

The dynamic model tracked the density of MDV over time in each isolator. In this model it is assumed:

1) After MDV infection, there is a delay before virus is first shed [31]. This is termed the primary latent period.

2) After this primary latent period, virus is shed at a constant rate (the primary viral shedding rate, measured in VCN per mg dust) for a set period of time, termed the secondary latent period.

3) Once this secondary latent period is over, virus is shed at a constant rate (the secondary viral shedding rate) until the termination of the experiment.

Additionally, the density of virus (VCN per mg dust) was calculated at the end of each day and any removal of birds was assumed to occur at the start of the day. While all parameters are assumed to be constant across all the birds in a single isolator, they can vary between isolators.

There are therefore four parameters estimated per isolator: the primary/secondary latent periods (in days) and the primary/secondary rates of viral shedding (VCN/mg dust). The primary and secondary latent periods are denoted by *T*^1 ^and *T*^2 ^respectively. Birds shed virus at a rate, *a*^1 ^(VCN per mg dust) from *T*^1^+1 to *T*^1^+*T*^2 ^days post infection; and at a rate *a*^2 ^(VCN per mg dust) from *T*^2^+1 until the end of the experiment (at 8 weeks post infection). The density of MDV in the dynamic model could be compared to the amount of virus recorded in the data (sampled every 7 days). We therefore estimated $a_{j}^{1}\left(v\right)$, $a_{j}^{2}\left(v\right)$, $T_{j}^{1}\left(v\right)$ and $T_{j}^{2}\left(v\right)$ for each virulence score *v *∈ {16.5, 36, 46} and each vaccine status *j *∈ {*Sham*, *HVT*, *Bivalent*}.

The model accounts for the removal of birds given in the mortality data and the number of complete hourly air changes (varied as a parameter *α*). The daily quantity of dust shed per bird was calculated by fit ting a cubic spline to dust MDV data provided from the same experiment [30].

To calculate the measurement error of a quantity of virus in a sample of dust we noted that a final MDV density was reached some time before the end of the sampling period. Such data can therefore be assumed to be identically and independently distributed. If *Y*_1 _and *Y*_2 _are samples from the experiment when the virus density and the data are lognormally distributed then:

$$\begin{array}{c} \;logY_{1},logY_{2}\sim N\left(\mu ,\sigma^{2}\right)\\ \Rightarrow logY_{1}-logY_{2}\sim N\left(0,2\sigma^{2}\right) \end{array}$$

To find the time point after which the data is assumed to have plateaued, four sets of points were examined: weeks 5, 6, 7 and 8. The differences between the logged data of weeks 7-8 provided the highest probability of both being drawn from a normal distribution (*p *= 0.8, Anderson-Darling *n *= 18) and that the distribution has a mean of zero (*p *= 0.40). The resulting standard deviation of the difference between log-transformed data was estimated to be 0.33 logVCN/mg dust.

Posterior distributions were found by means of McMC realisations by calculating the likelihood of the data given the parameter values, with lognormal errors of *σ*^2 ^= 0.33^2^/2. The burn-in time was set to 30,000 iterations and the posterior distribution was taken as every 10th sample from the following 90,000 iterations [37,38].

## Competing interests

The authors declare that they have no competing interests.

## Authors' contributions

KEA performed the statistical analysis and drafted the manuscript. AFR was involved in the drafting and revision of the manuscript for intellectual content. NJS was involved in the Bayesian statistical analysis. SWB and KGR were involved in supplying data and associated technical advice. SWB was involved in the revision of the manuscript for intellectual content. MEJW was involved in design of the statistical analysis and the drafting and revision of the manuscript for intellectual content. All authors approved the final manuscript.
